# Supplementary material for: Socio-Economic Position, Cancer Incidence and Stage at Diagnosis: A Nationwide Cohort Study in Belgium
Source: Cancers (Basel). 2021 Feb 24;13(5):933. doi: 10.3390/cancers13050933 (PMC7956180; doi:10.3390/cancers13050933)
Supplement: Supplementary file 1 [file cancers-13-00933-s001.zip › cancers-1113880-supplementary xml/Figure S1.pdf]

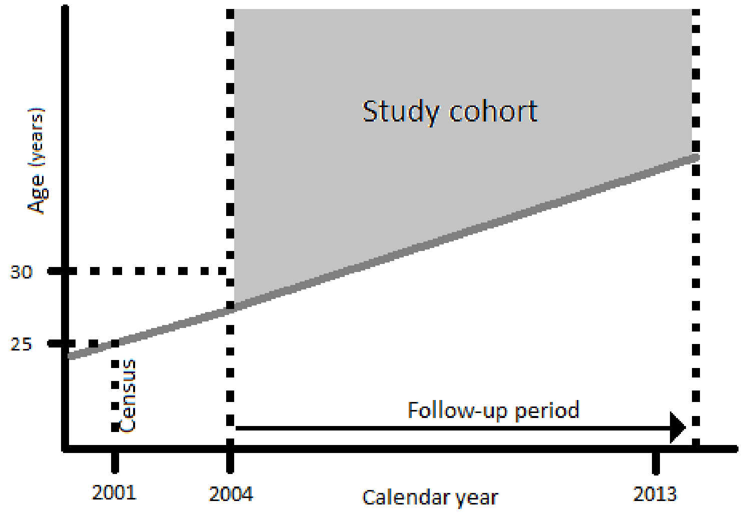

Figure S1. Definition of the observation period and study population by calendar time and age at time of census (1 October 2001) for cancer incidence analyses (2004–2013). Age-standardized incidence rates (ASR) are left-truncated below 30 years. All individuals aged 25 years or more at census were included in the cohort. However, they will only contribute to the ASR when they reach 30 years of age, which can be after start of the observation period, i.e. 1 January 2004. Follow-up is until 31 December 2013. There is no upper age limit.
